# Denoising Phase-Unwrapped Images in Laser Imaging via Statistical Analysis and DnCNN

**DOI:** 10.3390/mi15111372

**Published:** 2024-11-14

**Authors:** Yibo Xie, Jin Cheng, Shun Zhou, Qing Fan, Yue Jia, Jingjin Xiao, Weiguo Liu

**Affiliations:** 1 School of Optoelectronic Engineering, Xi’an Technological University, Xi’an 710021, China; 13319215096@163.com (Y.X.); zsemail@126.com (S.Z.); 2 Institute for Interdisciplinary and Innoation Research, Xi’an Technological University, Xi’an 710021, China; 3 Wuxi V-Sensor Technology Co., Ltd., Wuxi 214101, China; cks_qfan@163.com (Q.F.); 18205254782@163.com (Y.J.); 18115186192@163.com (J.X.)

**Keywords:** denoising, laser imaging, phase unwrapped, neural network, point cloud

## Abstract

Three-dimensional imaging plays a crucial role at the micro-scale in fields such as precision manufacturing and materials science. However, image noise significantly impacts the accuracy of point cloud reconstruction, making image denoising techniques a widely discussed topic. Statistical analysis of laser imaging noise has led to the conclusion that logarithmically transformed noise follows a Gumbel distribution. A corresponding neural network training set was developed to address the challenges of difficult data collection and the scarcity of phase-unwrapped image datasets. Building on this foundation, a phase-unwrapped image denoising method based on the Denoising Convolutional Neural Network (DnCNN) is proposed. This method aims to achieve three-dimensional filtering by performing two-dimensional image denoising. Experimental results show a significant reduction in the Cloud-to-Mesh Distance (C2M) statistics of the corresponding point clouds before and after planar filtering. Specifically, the statistic at 97.5% of the 2σ principle decreases from 0.8782 mm to 0.3384 mm, highlighting the effectiveness of the filtering algorithm in improving the planar fit. Moreover, the DnCNN method exhibits exceptional denoising performance when applied to real-world target data, such as plaster statues with complex depth variations and PCBs made from different materials, thereby enhancing accuracy and reliability in point cloud reconstruction. This study provides valuable insights into phase-unwrapped image noise suppression in laser imaging, particularly in micro-scale applications where precision is critical.

## 1. Introduction

**Figure 1:**
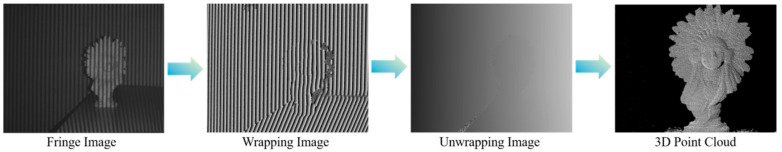
Image process of laser imaging reconstruction.

In 3D imaging research and applications, image accuracy and reliability are challenged by various noise sources. These noise sources can be categorized as detector noise, atmospheric noise, system noise, and coherent noise [1]. Of these, coherence noise is particularly significant and affects the accuracy of 3D data by degrading the sharpness of the images. Laser imaging is based on the principle that an observable fringe pattern is formed on the surface of an object by a laser projector, and the laser fringe image is captured by an imaging device, which generates a point cloud using 3D laser reconstruction techniques [2]. This process involves phase wrapping the image to record the fringes’ phase changes and resolving this information through phase-unwrapping techniques. The internal and external parameters of the camera are obtained based on Zhang’s camera calibration method [3], which is further mapped to determine the distance information of the object surface to generate the 3D point cloud, as shown in Figure 1. However, coherent noise may introduce non-negligible errors in laser fringe images, phase-wrapped images, phase-unwrapped images, and point cloud data [4], especially in phase-unwrapped images. Since an ideal phase-unwrapped image is spatially continuous and has a predictable trend [5], noise interference may lead to discontinuous jumps and anomalous phase variations, affecting the final 3D point cloud data. Therefore, denoising of the phase-unwrapped images is required to estimate and minimize the effect of noise on the data [6].

In recent decades, image denoising has been a focal point of research in the field of 3D imaging, and researchers have proposed a variety of filtering techniques, which can be broadly categorized into three main groups: spatial domain filtering, transform domain filtering, and deep learning-based filtering [7]. Among model-based approaches, BM3D [8] and WNNM [9] have effectively managed various noise levels. Afterwards, the advent of deep learning spurred significant interest in neural network-based denoising models. In 2017, Zhang K. introduced the DnCNN [10], which utilizes residual learning for blind Gaussian denoising, effectively handling unknown noise levels. This was followed by FFDNet [11], which modified the input to include down-sampled sub-images and generated noise-level images, enhancing adaptability and computational efficiency. In 2018, Guo S. proposed CBDNet [12], which advanced generalization by incorporating a user-defined noise intensity parameter, σ, and leveraging a combination of synthetic and natural noise data. The following year, Anwar S. introduced RIDNet [13], designed to capture intricate noise features through a multilevel residual block structure. By 2020, Wang Y. presented PMRID [14] at CVPR, an elegant algorithm that employs a compact network architecture, addressing the robustness of small networks against varying gain noises using a k-sigma transform. Most recently, in 2024, Chen Y. proposed a high-quality self-supervised image denoising method based on SDDW-GAN and CHRNet [15], which effectively removes noise through adversarial learning and convolutional neural networks.

Although deep learning filtering algorithms continue to emerge, their performance relies to some extent on high-quality training data, most of which are derived from image datasets such as the Berkeley Segmentation Dataset (BSD) [10,11,12,13,14,15]. However, existing BSDs are usually not sufficiently suitable for specific filtering tasks, and creating datasets suitable for specific filtering tasks is both complex and time-consuming. Therefore, this paper develops specialized neural network datasets to address this challenge.

In order to design algorithms that can effectively suppress noise, it is first necessary to have an in-depth understanding of noise type and its properties. Common types of noise include Gaussian, Rayleigh, Gamma, Salt Pepper, and Gumbel noise, each of which has its unique probability density function. Although researchers have assumed that the properties of the noise distribution in 3D imaging roughly conform to a Gaussian distribution [16], practical observations have shown that the noise distribution does not fully conform to the Gaussian model [17]. Therefore, to more accurately characterize the noise distribution, we perform a careful statistical analysis with the expectation of finding a noise model that is more consistent with real-world data.

The main contributions of this paper can be summarized as follows:

(1) This study provides an in-depth statistical analysis of the noise during 3D imaging. Through theoretical derivation, we determine that the noise after logarithmic transformation follows a Gumbel distribution, and the Gumbel fitting is performed on the noise data in the actual phase-unwrapped images to deepen understanding of the noise properties.
(2) By analyzing the properties of the actual phase-unwrapped image, we simulate and emulate the noiseless phase-unwrapped image to construct the corresponding datasets. In the simulated phase-unwrapped images, we introduce geometries with different sizes and positions to simulate the actual target and add tunable pixel steps, different pixel jumps, etc., to express the complexity of the actual imaging.
(3) Due to the effectiveness of the CNN in enhancing the capability and flexibility of image feature processing, we adopt the DnCNN model for laser denoising. In the self-built dataset, we introduce Gumbel noise at different scales to fully simulate the noise characteristics in laser imaging. Experimental verification of the actual phase-unwrapped images and an analysis of the corresponding 3D reconstructed point clouds demonstrate the effectiveness and robustness of the DnCNN model for practical applications in laser denoising.

The structure of this paper is as follows: Section 2 accurately describes the types of noise in 3D imaging using theoretical analysis and experimental validation. Section 3 describes the network architecture, the characteristics of the phase-unwrapped image, and how to build up the image datasets. Section 4 demonstrates the experimental evaluation of noise suppression in phase-unwrapped images and verifies the filtering effect with the corresponding reconstructed point clouds.

## 2. Laser Imaging Noise Analysis

### 2.1. Noise Statistical Modeling

**Figure 2:**
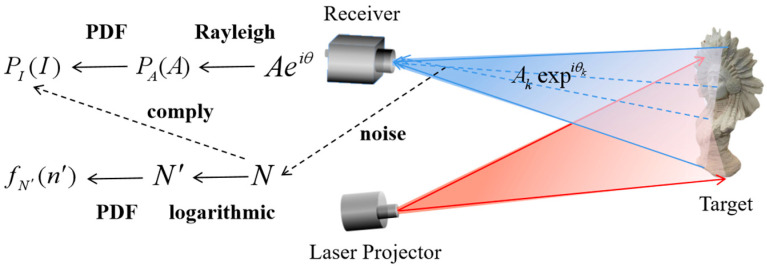
Schematic of the theoretical analysis of laser imaging.

In 3D imaging, noise analysis is critical to ensure imaging quality and accuracy. In order to effectively suppress noise and improve imaging accuracy, an in-depth study of the statistical properties in phase-unwrapped image noise is needed to construct more accurate noise models. When a light pulse emitted from a light source propagates in multiple directions and encounters an irregular scatterer or inhomogeneous interface, the scattered light waves will be generated in different directions from the original propagation. Some scattered light waves return as backscatter and are captured by the receiver. Since these returned scattered light waves come from the same light source, they follow specific statistical regularity [18]. A theoretical analysis of laser imaging is presented in Figure 2.

In an imaging system, the echo generated by each scatterer can be expressed as a complex number, $A\exp^{i\theta_{k}}$, and the total received echo signal is viewed as a superposition of multiple echoes, represented by Equation (1):

(1) $$Ae^{i\theta }=\sum_{k=1}^{n}A_{k}\exp^{i\theta_{k}}$$

where $A_{k}\exp^{i\theta_{k}}$ denotes the echo at the *k*th scattering point; *A_k_* and *θ_k_* are amplitude and phase, respectively; and *n* is the total number of scattering points. According to the laser imaging principle [19], its measurement signal is composed of pairs of received signals in the in-phase and orthogonal channels (*A*cos*θ* and *A*sin*θ*) weighted by the point-target spread function [20]. The total echo signal can also be expressed as a complex number $Z=A_{r}+jA_{i}=A\cos\theta +jA\sin\theta$. When *n* is large, *A_r_* and *A_i_* approximately conform to a Gaussian distribution with mean 0 and standard deviation *σ* according to the central limit theorem [21]. The amplitude *A* of the echo signal is calculated by Equation (2):

(2) $$A=\left|Z\right|=\sqrt{A_{r}^{2}+A_{i}^{2}}$$

where *A_r_*^2^ and *A_i_*^2^ obey a chi-square distribution with freedom degree 1. According to the nature of the chi-square distribution, the square root of the sum of two independent chi-square distributed random variables obeys the Rayleigh distribution [22]. Therefore, the probability density function of the echo signal amplitude conforms to the Rayleigh distribution, which is represented by Equation (3):

(3) $$P_{A}(A)=\frac{A}{\sigma^{2}}\exp\left(-\frac{A^{2}}{2\sigma^{2}}\right)$$

where *σ* is the standard deviation. And the pixel intensity *I* can be calculated by *I* = *A*^2^ and the probability density function of *I* is expressed as Equation (4):

(4) $$P_{I}(I)=P_{A}(A=\sqrt{I})\left|\frac{dA}{dI}\right|=\frac{1}{2\sigma^{2}}\exp\left(-\frac{I}{2\sigma^{2}}\right)$$

We know that the coherence between lasers generates a large amount of multiplicative noise [23,24], which is modelled as Equation (5):

(5) G(x,y)=F(x,y)N(x,y)

where *G*(*x*,*y*) is the laser noise image, *F*(*x*,*y*) is the noiseless ideal image, and *N*(*x*,*y*) is the noise component. In order to simplify the noise model and enhance the stability of the statistical analysis, we use a logarithmic transformation to convert the multiplicative noise into the additive noise [25], as seen in Equation (6):

(6) $$G^{\prime }(x,y)=F^{\prime }(x,y)+N^{\prime }(x,y)$$

where *G′*(*x*,*y*), *F′*(*x*,*y*)*,* and *N′*(*x*,*y*) are the laser noise image of the logarithmic transformation, the noiseless ideal image of the logarithmic transformation, and the noise component of the logarithmic transformation, respectively. We can assume that *N′* has the form

(7) $$N^{\prime }=a\ln(N)+b$$

where *a* and *b* are the coefficients; then, the probability density function of *N′* is obtained by transforming the probability density function of the original noise *N*:

(8) $$f_{N^{\prime }}(n^{\prime })=\frac{f_{N}(n)}{\left|\frac{dn^{\prime }}{dn}\right|}$$

where

(9) $$\frac{dn^{\prime }}{dn}=\frac{a}{N}$$

Based on Equation (7), Equation (10) is obtained:

(10) $$N=\exp^{\frac{N^{\prime }-b}{a}}$$

During imaging, noises usually originate from the superposition of many scattered light waves produced by the same light source, so they follow the same statistical laws as the echo signal. Based on this, we can obtain the probability density function of the original noise:

(11) $$f_{N}(n)=\frac{1}{2\sigma^{2}}\exp\left(-\frac{\exp^{\frac{n^{\prime }-b}{a}}}{2\sigma^{2}}\right)$$

Finally, the probability density function of additive noise is obtained by organizing Equations (8) and (11) as Equation (12):

(12) $$f_{N^{\prime }}(n^{\prime })=\frac{1}{2a\sigma^{2}}\cdot \exp^{\frac{n^{\prime }-b}{a}}\cdot \exp\left(-\frac{\exp^{\frac{n^{\prime }-b}{a}}}{2\sigma^{2}}\right)$$

Through the above theoretical analysis, we find that the probability density function of the original noise distribution after logarithmic transformation conforms to the Gumbel distribution [26], which accurately establishes the noise model in laser imaging.

### 2.2. Noise Model Validation

To investigate the system noise characteristics further, in this section, we experimentally verify that the image noise distribution in a 3D imaging system follows the Gumbel distribution. Plane and wall are selected as target objects, and their simple geometric properties and controllable surface reflection properties provide ideal test conditions for the verification process. The corresponding phase unwrapped image is obtained through the three-dimensional scanning of the object, and their noise distributions are analyzed in detail.

**Figure 3:**
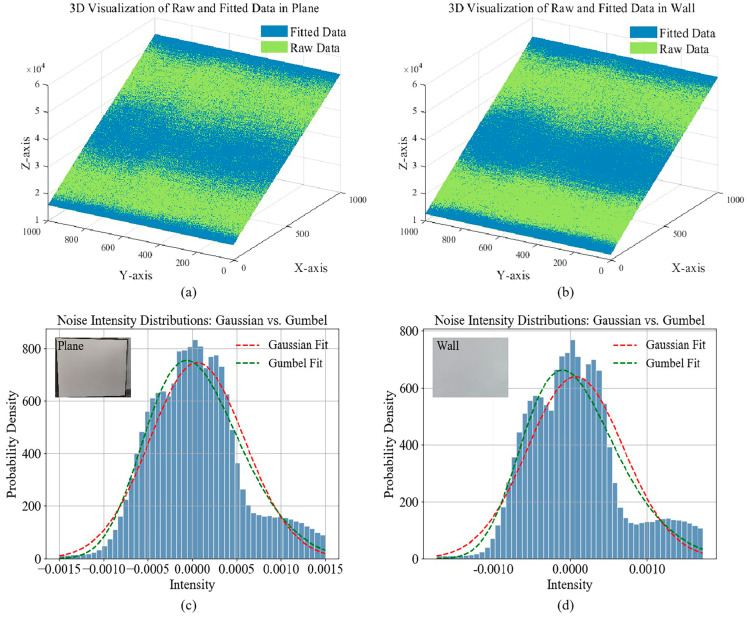
Noise distribution of different targets: (**a**) 3D display of raw data and fitted data for the plane; (**b**) 3D display of raw data and fitted data for the wall; (**c**) noise intensity distribution for the plane; (**d**) noise intensity distribution for the wall.

Firstly, we reasonably assume that the actual phase-unwrapped images contain mainly noise components and functional phase change signals. For this purpose, a polynomial equation is fitted to it using a least-squares optimization algorithm, as shown in Equation (13):

(13) $$Z=\alpha_{0}+\alpha_{1}X+\alpha_{2}Y+\alpha_{3}X^{2}+\alpha_{4}XY+\alpha_{5}Y^{2}+\alpha_{6}X^{3}+\alpha_{7}X^{2}Y+\alpha_{8}XY^{2}+\alpha_{9}Y^{3}$$

where *Z* is the dependent variable, *X* and *Y* are the independent variables, and *α_k_* are the model parameters. We consider that the fitted plane represents the noiseless proper signal, as shown in Figure 3a,b, where the blue plane is the polynomial fitted data, and the green plane is the three-dimensional display of the original data. Subsequently, according to Equation (6) of the additive noise model, after logarithmic transformation of the original data and fitted data, we subtract point by point to extract the noise components, as defined by Equation (14):

(14) log[N(x,y)]=log[G(x,y)]−log[F(x,y)]

After cleaning the data based on the inter-quartile range (IQR) method [27], we perform a statistical analysis of the noisy data using the maximum likelihood estimation (MLE) algorithm [28] and fit the Gaussian and Gumbel distributions. By analyzing the fitted curves and histograms of the models, as shown in Figure 3c,d, we found that the Gaussian distribution exhibits a classical symmetric bell-shaped curve. In contrast, the Gumbel distribution shows an apparent skewness with a longer tail and slower decay rate. This property makes the Gumbel distribution show higher fitting accuracy when describing the actual data distribution. It verifies the consistency between the log-transformed laser noise data and the Gumbel distribution.

To assess the fit effectiveness of the data distribution, the Kolmogorov–Smirnov (K-S) test method [29] was used, which measures goodness of fit by calculating the maximum absolute difference between the empirical distribution function (EDF) of the sample data and the cumulative distribution function (CDF) of the reference distribution. The statistic for the K-S test is defined by Equation (15):

(15) $$D=\max\left\{EDF(x)-CDF(x)\right\}$$

where a more minor *D* indicates a higher agreement between the empirical and cumulative distributions. The K-S test results for the Gaussian and Gumbel distributions show that the K-S of the plane region is 0.0375 and 0.0292, and that of the wall region is 0.0520 and 0.0358, respectively. This suggests that the Gumbel distribution has higher fitting accuracy in describing the noise of the plane and the wall, which validates its superiority in simulating laser image noise modelling. Thus, this section confirms the applicability of the Gumbel distribution in the noise characterization of 3D imaging systems and provides strong support for the research and application of related noise models.

## 3. DnCNN Model for Phase-Unwrapped Image Denoising

In this section, we will briefly introduce the Denoising Convolutional Neural Network (DnCNN)’s architecture and analyze the properties of phase-unwrapped images. With well-designed parameter tuning, we construct an ideal training set of phase-unwrapped images to use the DnCNN to learn the residuals between noisy and noiseless images to effectively remove noise and thus improve the accuracy and reliability of the subsequent 3D reconstruction processing. Based on the analysis in Section 2, we confirm that the noise in 3D imaging follows a Gumbel distribution. To simulate the noise environment in real laser images more realistically, we introduce different Gumbel noise levels in the multiple phase unwrapped images. Such a dataset construction strategy will show more adaptability and robustness in future applications.

### 3.1. DnCNN Architecture

**Figure 4:**
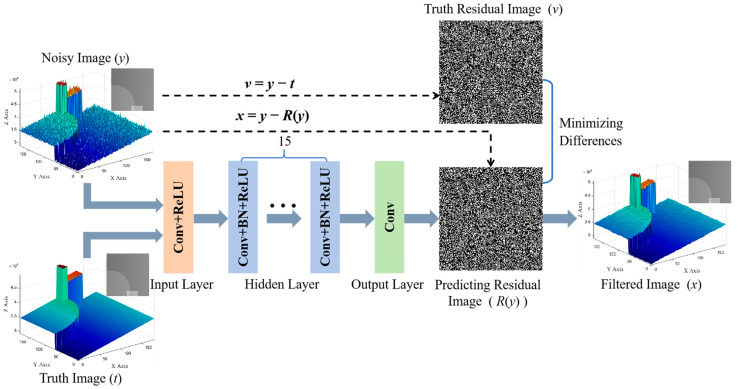
Schematic diagram of DnCNN architecture.

This paper uses a DnCNN to suppress noise in phase-unwrapped images. Figure 4 illustrates the architecture of the DnCNN, which is designed with a depth of 17 layers to capture rich spatial features. The architecture contains three principal components:

(1) The input layer employs convolutional layers and ReLU activation functions to extract initial image features. This layer has 64 convolutional kernels that generate 64 distinct feature channels.
(2) The hidden layer further refines and integrates the image features through convolutional layers, batch normalization layers, and ReLU activation functions. All these layers utilize 3 × 3 convolutional kernels to enhance feature extraction.
(3) The output layer employs the convolutional layer to combine 64 feature channels to reconstruct the filtered image linearly.

Each convolutional layer is accompanied by a ReLU activation function, which introduces nonlinearity to enhance the model’s ability to learn complex features. The batch normalization process in the hidden layer effectively reduces the internal covariate bias and accelerates the convergence of the model. The architecture is designed to optimize the noise suppression effect during phase unwrapping and enhance the 3D imaging quality.

The network training objective is to learn a residual mapping *R*(*y*), where *y* is an image with noise so that the residual image *R*(*y*) predicted by the network is close to the real residual image *v* (*v* = *y* − *t*, *t* is the Truth Image). Finally, the ideal image *x* is obtained by *x* = *y* − *R*(*y*), as shown in Figure 4. During the training process, the mean square error (MSE) is used as a loss function to optimize the residual mapping, which is represented by Equation (16):

(16) $$MSE=\frac{1}{N}\sum_{i=1}^{M}{\left(v_{i}-R_{i}(y_{i})\right)}^{2}$$

where *M* is the number of training samples; *v_i_* is the true residual of the *i*th phase-unwrapped image; and *R_i_*(*y_i_*) is the residual result predicted by the network. The learning rate is dynamically adjusted using the Adam optimizer with an initial learning rate of 1 × 10^−3^, reduced to about 1 × 10^−4^ after training.

### 3.2. Building the DnCNN Training Set

**Figure 5:**
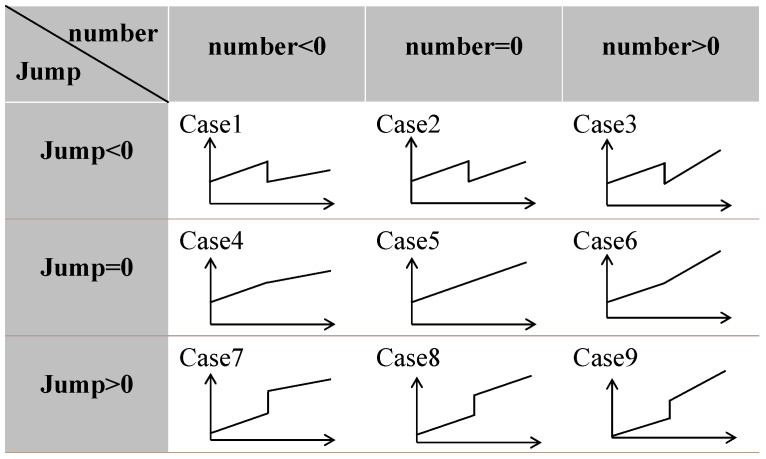
Pixel distribution type of phase unwrapped image.

The dataset we constructed contains 400 high-depth (16-bit) phase-unwrapped images; these high-precision data help to improve the algorithm’s sensitivity to image noise and, thus, the accuracy of reconstruction. The image size of the dataset is 180 × 180 pixels. Based on the analysis of the actual phase-unwrapped image, the pixel values increase from left to right in steps of 30–50, so the pixel step range of the simulated phase-unwrapped image is set to Step ∈ [30, 50] to simulate the increasing trend of the pixel. Meanwhile, considering the depth difference of the target object, there will be a pixel jump in the image, which is set to be in the range of Jump ∈ [−30,000, 30,000] to reflect the pixel mutation due to the different target depths. In addition, due to the non-parallel nature of the actual target concerning the reference plane, we add a random number with a range of number ∈ [−100, 100] to the original phase tilt to simulate the uncertainty of the target phase change. This design makes the phase variation appear random so that our dataset can more genuinely reflect the noise characteristics in the 3D imaging process and provides a rich data source for training the DnCNN. By varying different parameters, the pixel distribution of the phase unwrapped image was summarized into nine types, as shown in Figure 5.

**Figure 6:**
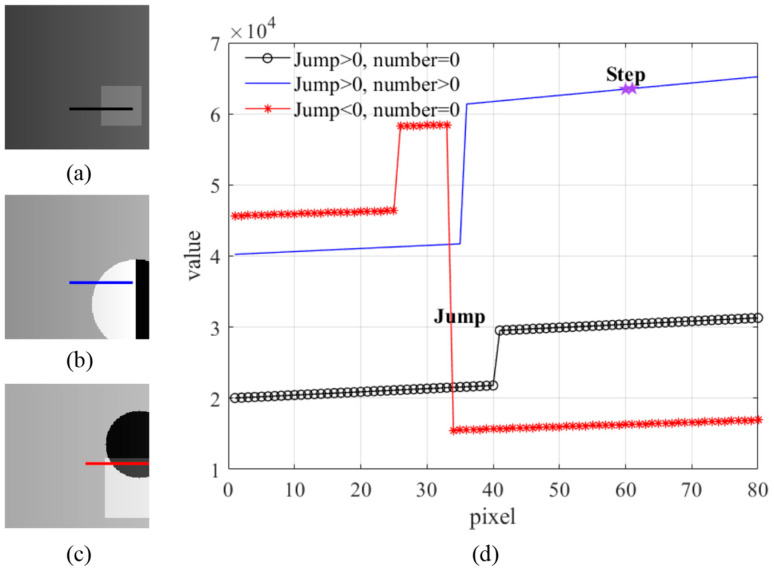
Pixel distribution of simulated phase-unwrapped images: (**a**) phase-unwrapped image of a square target; (**b**) phase-unwrapped image of a circular target; (**c**) phase-unwrapped image of a combined square and circular target; (**d**) local light intensity distribution of the simulated phase-unwrapped images.

In order to enhance the generalization capability and adaptability of the DnCNN in image denoising tasks, we introduce diverse geometries, including squares and circles with different sizes and positions in the phase-unwrapped image dataset. These shapes are set as target objects to simulate the complex scenes encountered during the imaging process. As shown in Figure 6a–c, several data patterns used for training are demonstrated, reflecting the pixel variations under different Jump and number conditions. We selected the light intensity distributions of local regions from the images in Figure 6a–c and analyzed the different pixel variations, as seen in Figure 6d, where the *x*-axis represents the pixel position, and the *y*-axis represents the corresponding pixel value. The Jump parameter reflects whether the pixel of the target increases or decreases relative to the pixel of the reference plane in the phase unwrapped image; the number parameter’s variation represents the randomness of the target’s pixel tilt concerning the reference plane, and the Step parameter describes the value difference between neighboring pixel positions. Specifically, in Figure 6d, the phase intensity distribution of the first pattern has only one positive Jump; the second pattern introduces a positive phase tilt on top of the positive Jump; and the light intensity distribution of the last pattern contains one upward and one downward Jump. The light intensity distributions of these modes cover almost all image types in the training dataset, fully demonstrating the diversity and complexity.

Facing the challenge of Gumbel-distributed noise, we set the noise scale parameter range *σ* ∈ [0.001, 0.01] to cover a variety of noise levels that may be encountered in real imaging. For model training, we chose a patch size of 40 × 40 pixels as the training unit and cropped multiple 128 × 1600 pixel patches from the image. The model is designed to suppress Gumbel noise in phase-unwrapped images and achieve more accurate data reconstruction in 3D imaging.

We built a test dataset consisting of two parts: simulated phase images with added noise and phase-unwrapped images from a real laser imaging system. Note that all test images were not included in the training dataset to ensure the robustness of the test results.

## 4. Real Laser Noise Suppression Evaluation

In recent years, neural network-based phase image filtering techniques have gained much attention. For example, Yan K. proposed a deep learning-based algorithm for noise reduction in fringe patterns [30]. Liu B. proposed a deep learning model called In-CNN, which is specialized for denoising interferometric phase images [6]. Li X. proposed an attention wavelet residual denoising network based on a multilevel wavelet convolutional neural network [31], which utilizes the properties of wavelet transform and combines attention force mechanism and residual learning to remove speckle noise effectively. The above research mainly focuses on filtering fringe images in laser imaging. However, the research in this paper focuses on filtering phase-unwrapped images in laser imaging. Through theoretical analysis and experimental verification, this paper first establishes the corresponding noise model and profoundly analyzes the noise characteristics of phase-unwrapped images. Based on this, this paper constructs a specific training set. It filters different types of actual phase images to further verify the effectiveness of the DnCNN algorithm in removing specific noises.

### 4.1. Experimental Program

**Figure 7:**
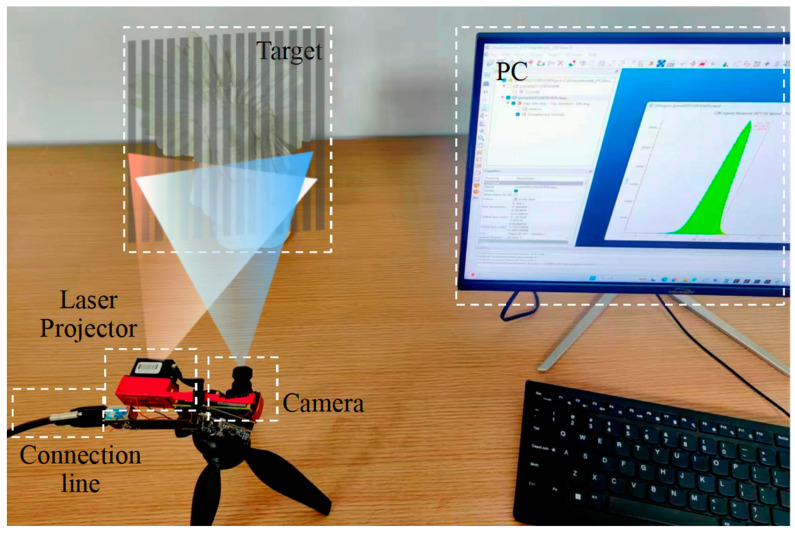
Actual scene of the laser image acquisition system.

The actual scene of the laser image acquisition system is shown in Figure 7, including the 3D structured light imaging system, the target, and the PC. The red and blue lights in the figure indicate the MEMS laser’s scanning range and the camera’s acquisition range, respectively. The parameters of the structured light projection module (provided by Wuxi V-Sensor Technology Co., Ltd., Wuxi, China) are as follows: the light source has a wavelength of 850 ± 5 nm, a power output of 1.5 W, and an optical resolution of 900 × 1. The camera (provided by Wuxi V-Sensor Technology Co., Ltd., Wuxi, China) specifications include a resolution of 1280 × 1024 pixels and a focal length of 6mm. These parameters are further detailed in Table 1. The experimental environment for this paper was set up in Python3.7, leveraging the PyTorch1.8 framework for network realization. It was conducted on a system equipped with an Intel Core i7-10750H CPU and an Nvidia GeForce GTX GPU.

### 4.2. Quantitative and Qualitative Analysis

In this section, we focus on analyzing the features of the phase unwrapped images, with particular attention to the fluctuations in light intensity [32], to verify the effectiveness of the DnCNN filtering algorithms in reducing noise. We aim is to improve the quality of the 3D point cloud through the filtering process of the 2D image. For this purpose, we reconstructed the phase-unwrapped images before and after filtering into the corresponding point clouds to observe point fluctuations in the point clouds. In evaluating the quality of the planar point cloud, the Cloud-to-Mesh Distance (C2M) method [33] is used to fit the point cloud to an optimal plane and the normal vector of this plane is used to compute the distances between the z-coordinates of the points and the fitted plane. The distribution of these distances is demonstrated by constructing histograms, and the farthest distance value of the C2M at cumulative 97.5% was used as a critical metric to assess the overall fitting quality of the planar point cloud. In summary, this study comprehensively compares and analyzes the performance of the DnCNN filtering algorithm in laser imaging noise suppression at both 2D image and 3D point cloud levels.

### 4.3. Experiments with Real Laser Data

In this study, we acquired laser images using a laser imaging system and obtained phase distribution by phase-unwrapping processing. To ensure the comprehensiveness and representativeness of the experiment, we selected three kinds of targets with different characteristics as experimental objects. First, the planar target was chosen to represent the case of a uniform surface, consistent depth, and simple structure, and the characteristics of this target made it a benchmark test that helped to evaluate the performance of the algorithms in processing a uniform surface. Second, as a complex target with a single material but with depth variations, the plaster statues can effectively simulate the diversity and complexity in a real scene, challenging the algorithm’s ability to handle images with subtle texture and depth variations. Finally, the printed circuit board (PCB), representing targets with different materials and varying refractive indices, further extend the breadth of the experiment to test the algorithm’s performance under complex optical properties. By testing these three objectives, we can cover most real-world application scenarios and provide solid experimental support for the robustness of the DnCNN algorithm.

#### 4.3.1. Smooth Surface Experiment

**Figure 8:**
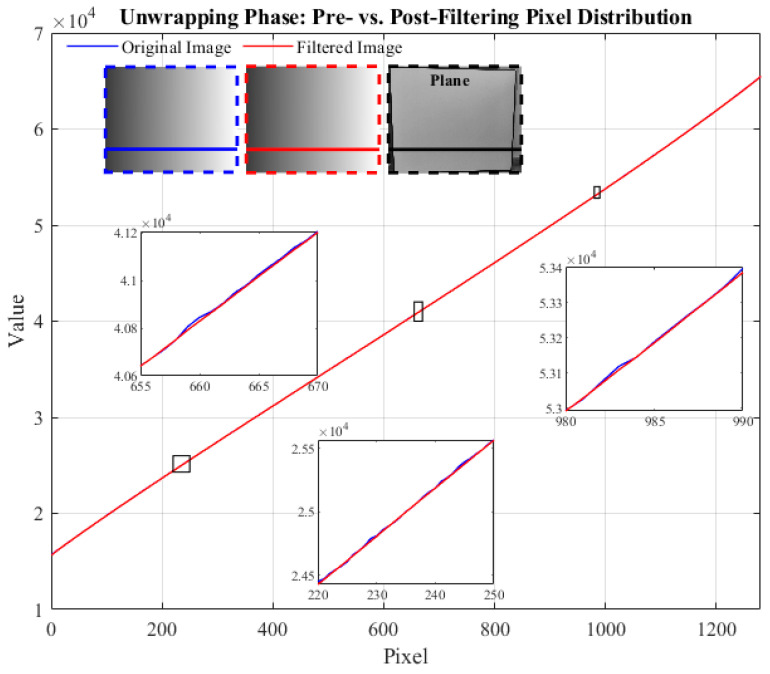
Pixel distribution of the phase-unwrapped image before and after filtering for plane.

In this experiment, we select the plane as the target for a detailed analysis of the light intensity distribution in the phase-unwrapped image before and after filtering. Figure 8 shows the pixel distribution of a particular row in the image, where the blue represents the unfiltered original data, and the red represents the filtered image. By zooming in on a specific region, it becomes clear that the raw planar data exhibits small pixel fluctuations due to noise. These fluctuations visually create irregular undulations, leading to the degradation of the overall image quality. In contrast, the filtered image shows apparent smoothing features, with the lines becoming more stable and uniform. This change indicates that the noise has been effectively suppressed and reflects the filtering algorithm’s significant effect in improving the image quality.

**Figure 9:**
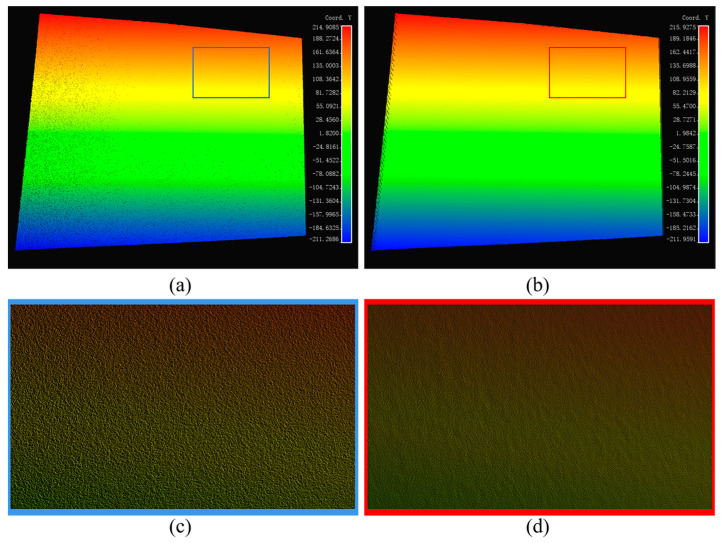
Reconstructed point cloud before and after filtering for plane: (**a**) point cloud before filtering; (**b**) point cloud after filtering; (**c**) ROI point cloud before filtering; (**d**) ROI point cloud after filtering.

When converting phase information into actual 3D coordinates, we employ Zhang’s camera calibration method to determine the camera’s internal and external parameters. This approach enables the extraction of the actual *z*-axis coordinates via the phase–height mapping relation. By incorporating the camera’s internal and external parameters, we can accurately locate each pixel in the x- and y-axes, thereby facilitating the reconstruction of a comprehensive 3D point cloud. Figure 9a,b display the point cloud data before and after filtering, respectively. We selected the areas highlighted by the blue and red boxes for closer inspection. In Figure 9c, the surface of the localized region of the original point cloud exhibits discernible granularity, which could negatively impact subsequent 3D reconstruction and measurement accuracy. In contrast, Figure 9d demonstrates that the filtered region exhibits significantly smoother surface details with noise removal, thereby confirming the effectiveness of the filtering algorithm.

**Figure 10:**
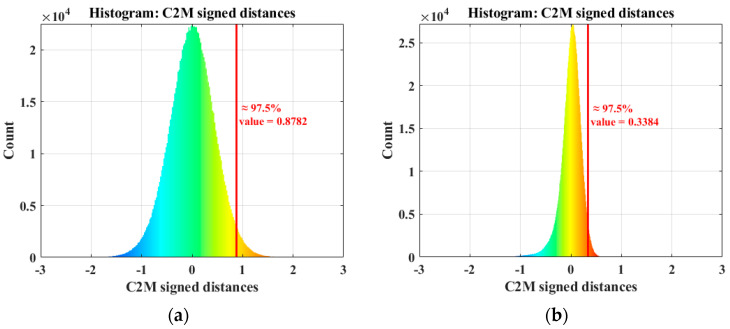
Cloud-to-Mesh Distance statistics of the point cloud: (**a**) Cloud-to-Mesh Distance statistics before filtering; (**b**) Cloud-to-Mesh Distance statistics after filtering.

The Cloud-to-Mesh Distance (C2M) metric was employed in this study to assess the fitting quality of the point cloud. Specifically, we measured the distance between the region of interest (ROI) in the point cloud and the surface of the fitted mesh. The distance values were symbolically labelled according to the normal direction of the mesh: points inside the mesh were assigned negative distance values, while points outside were assigned positive values. To visualize the distribution of these distances, histograms were plotted to compare the C2M distributions of the point cloud before and after filtering, as shown in Figure 10a,b. Statistical analysis reveals that in 97.5% of the points set in the original point cloud, the maximum distance is 0.8782mm. After processing with the DnCNN filtering algorithm, the maximum distance for 97.5% of the point set is significantly reduced to 0.3384mm. These results demonstrate that the applied filtering algorithm effectively reduces noise and enhances the fit of the point cloud to the plane.

#### 4.3.2. Plaster Statue Experiment

**Figure 11:**
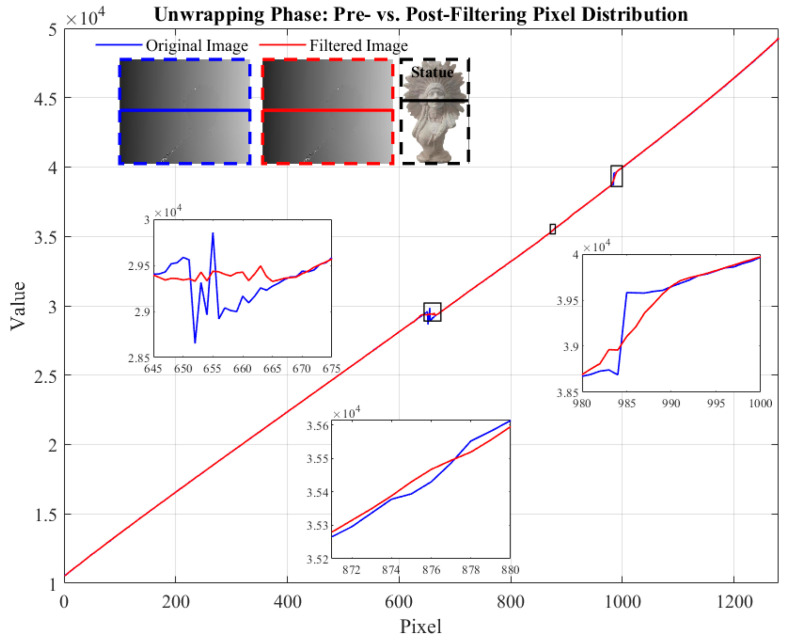
Pixel distribution of the phase-unwrapped image before and after filtering for plaster statue.

Using a complex plaster statue with rich surface details as a test target presents a challenge for the filtering algorithm. By analyzing the phase-unwrapped image light intensity distribution before and after filtering, as shown in Figure 11, we can accurately evaluate the filtering effect. At pixel positions of 645~645 and 980~1000, there are apparent jumps between the background light intensity and the target edge, indicating that the edge region is more affected by the sudden light change and that the noise step is significant. Despite the drastic changes in light intensity of the phase-unwrapped image, the DnCNN effectively suppresses the noise and reduces the pixel jumps. At a pixel position of 870~880, the light intensity distribution of the original image and the filtered image does not change much, indicating that the method preserves the detailed features of the plaster statues. After the filtering process, the light intensity distribution in the boundary region is smoother, the noise is effectively suppressed, and the target region realizes the smoothness of the surface while retaining the basic features, proving this filtering algorithm’s excellent performance in dealing with complex surfaces.

**Figure 12:**
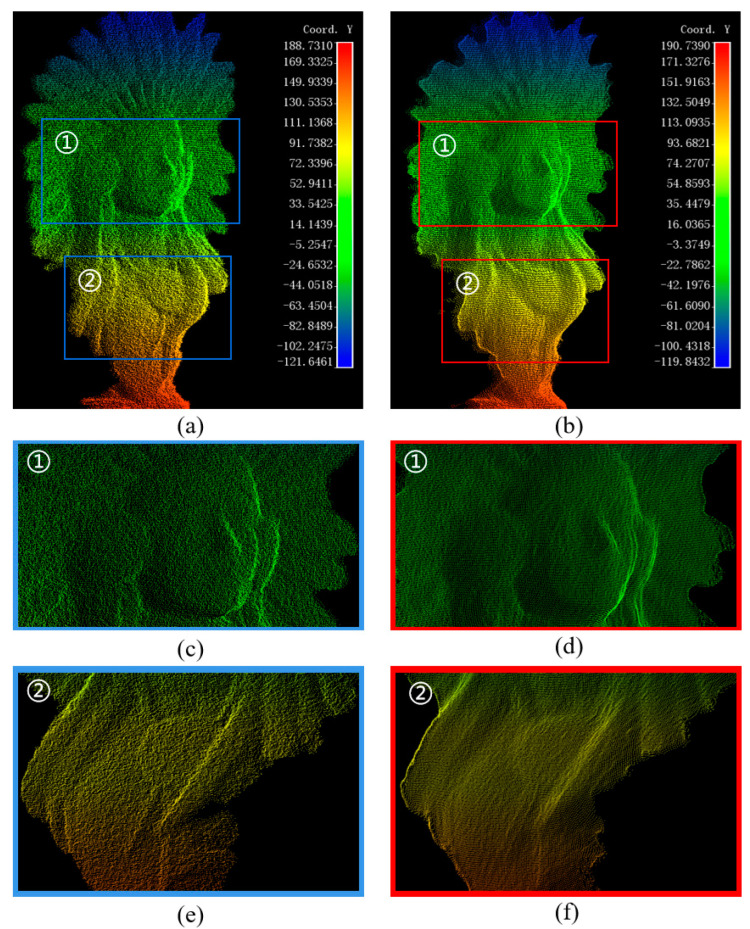
Reconstructed point clouds before and after filtering for plaster status: (**a**) point cloud before filtering; (**b**) point cloud after filtering; (**c**) ROI-1 point cloud before filtering; (**d**) ROI-1 point cloud after filtering; (**e**) ROI-2 point cloud before filtering; (**f**) ROI-2 point cloud after filtering.

Figure 12a,b shows that the reconstructed point clouds before and after filtering show significantly different results. In calculating the distance between neighboring points of the point cloud as 0.50 mm, we find that the accuracy is beyond the practical capability of the imaging system. Despite the texture information on the physical surface of the plaster statue, the system’s limitations fail to capture these details fully. Thus, the point cloud’s detailed information is noisy in subjective vision. The surface of the original point cloud presents as rough, cluttered, and visually poor, especially when zoomed in on a specific region of interest (ROI); as shown in Figure 12c,e, the noise is even more apparent, which mainly originates from the mutual interference of laser beams during the laser scanning process. In contrast, after DnCNN filtering, the noise of some pseudo-detail information is effectively suppressed, the surface texture of the point cloud is significantly smoothed, the cluttered points are effectively removed, and the overall surface features are more transparent and coherent, as shown in Figure 12d,f. This demonstrates the solid adaptive ability of the filtering algorithm to deal with complex target point cloud noise.

#### 4.3.3. PCB Experiment

**Figure 13:**
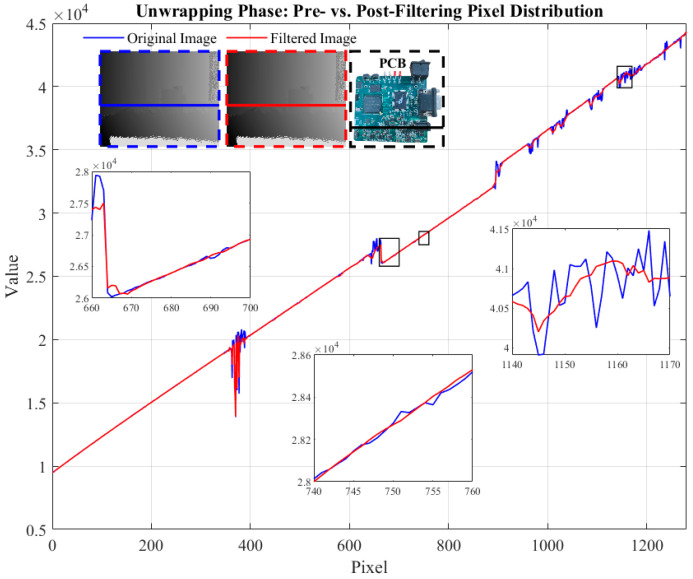
Pixel distribution of phase-unwrapped images before and after filtering for PCB.

In this experiment, we choose a printed circuit board (PCB) as the study object to evaluate the impact of different materials on the point cloud quality and the robustness of the DnCNN algorithm in the face of inhomogeneous reflectance properties. Since a PCB is usually composed of multiple materials, the reflectivity differences in various materials lead to different noise levels, affecting point cloud quality. To address this issue, we perform phase-unwrapping processing on the target and obtain the corresponding light intensity distribution, as shown in Figure 13. The results show that the DnCNN algorithm exhibits superior performance in handling Gumbel noise caused by different materials. It can effectively smooth the light intensity distribution, eliminate various complex noises, and is highly adaptable in multi-material environments, successfully coping with the noise challenges caused by material reflectivity differences.

**Figure 14:**
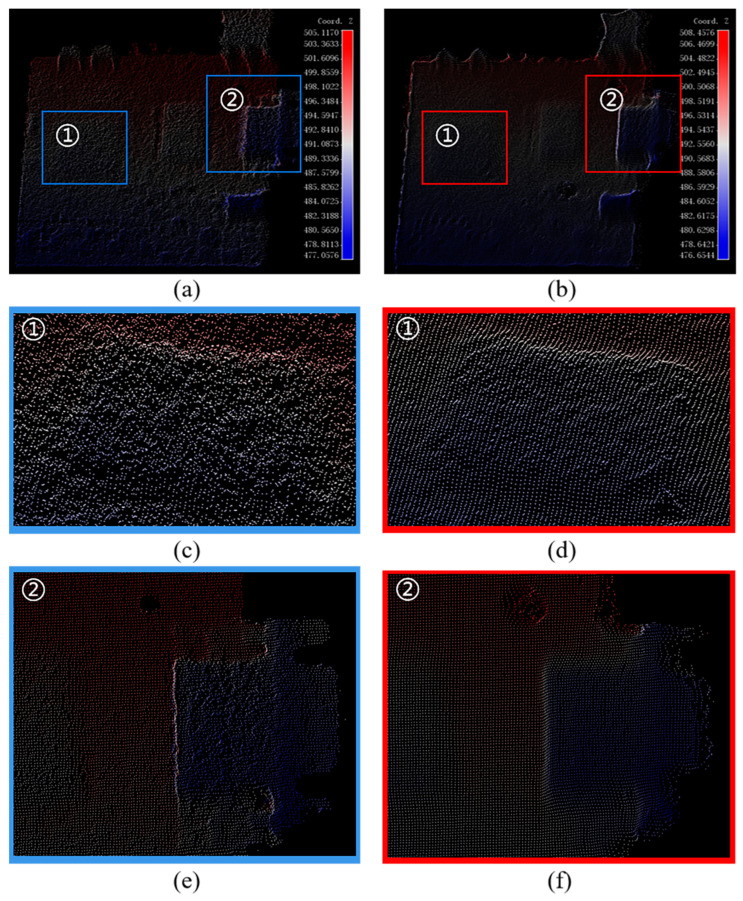
Reconstructed point cloud before and after filtering for PCB: (**a**) point cloud before filtering; (**b**) point cloud after filtering; (**c**) ROI-1 point cloud before filtering; (**d**) ROI-1 point cloud after filtering; (**e**) ROI-2 point cloud before filtering; (**f**) ROI-2 point cloud after filtering.

In order to deeply analyze the effects of different material noises on the object surface, we further compared the point cloud results before and after filtering, as seen in Figure 14a,b. In particular, we focused on critical areas of the PCB, such as chip areas and interface locations, as shown in Figure 14c,e. In the original point cloud, there are noise points of varying strength on the surface, significantly interfering with object recognition. The point cloud processed by DnCNN filtering, as shown in Figure 14d,f, not only retains the basic structural features of the PCB but also effectively eliminates the noise due to reflectivity differences. This indicates that the DnCNN filtering algorithm performs well in dealing with noise from different materials, and its significant filtering effect lays a good foundation for subsequent point cloud processing and object recognition.

This study introduces a dataset of phase-unwrapped images created using the DnCNN, applied to denoise 3D point clouds via 2D image filtering. Experiments were conducted on three target features, plane surface, plaster statue, and PCB, to evaluate the algorithm’s robustness across different materials and surface conditions. On flat surfaces, the DnCNN significantly reduced point cloud noise, improving surface smoothness and fitting accuracy. In plaster statue experiments, despite challenges due to light intensity variation, the DnCNN effectively removed excess noise, enhancing visual quality and detail retention. For PCB experiments, the DnCNN addressed noise caused by material reflectivity differences, preserving vital structural features. The results demonstrate the DnCNN’s potential for accurate environmental perception and object recognition in 3D modeling and autonomous driving. Additionally, the DnCNN can be applied in medical imaging to denoise and enhance CT scans, improving image quality and supporting more accurate diagnoses. In industrial manufacturing, particularly for products with complex surfaces and high precision (e.g., PCB boards, precision mechanical parts), DnCNN-based denoising can improve inspection accuracy and surface quality assessment.

## 5. Conclusions

In practical 3D imaging applications, noise significantly impacts micro-scale reconstruction results. To tackle this challenge, we developed a deep learning-based denoising model designed explicitly for laser imaging to address phase-unwrapped image noise. Through theoretical derivation and experimental validation, we thoroughly analyzed the laser imaging noise after logarithmic transformation, which follows a Gumbel distribution and is strongly supported in noisy data fitting. To simulate the complexities encountered in the imaging process, we constructed a phase-unwrapped image dataset encompassing multiple geometries, providing rich material for algorithm training and testing. The performance evaluation of the DnCNN filtering algorithm on objects made of various materials (e.g., plane, plaster statues, and PCBs) demonstrates that the DnCNN model effectively suppresses Gumbel noise, particularly in scenarios with varying levels of homogeneity, resulting in significantly improved point cloud quality. This study not only introduces a method for reducing laser imaging noise but also highlights the application potential of our approach in enhancing the imaging fidelity for different materials and geometries, paving the way for advancements in precision applications like micro-manufacturing. In industrial manufacturing, the method enhances the identification of subtle defects (e.g., cracks, scratches, irregular surfaces) and manufacturing defects, thus effectively supporting automated quality inspection and defect localization.

## Figures and Tables

**Table 1:** Parameters of laser-structured light module and infrared camera.

Items	Laser-Structured Light Module	Infrared Camera
Band/nm	850 ± 5	800~900
Resolution	900 × 1	1280 × 1024
Working distance/mm	600~900	manual focus
Pixel Size/μm	/	4.8
Focal Length/mm	/	6
Power/W	1.5	/

## Data Availability

The original contributions presented in the study are included in the article, further inquiries can be directed to the corresponding author.
